# The incidence and impact of ‘Tandem Neurotrauma’

**DOI:** 10.1016/j.bas.2023.102702

**Published:** 2023-10-21

**Authors:** Xiaoyu Yang, Benjamin M. Davies, Jonathan P. Coles, David K. Menon, Daniel J. Stubbs, Aref-Ali Gharooni, Wunna Aung, Michelle L. Starkey, Douglas Hay, Fahim Anwar, Ivan S. Timofeev, Adel Helmy, Virginia F.J. Newcombe, Mark R.N. Kotter, Peter J.A. Hutchinson

**Affiliations:** a Division of Neurosurgery, Department of Clinical Neurosciences, University of Cambridge, Cambridge, United Kingdom; b Department of Neurosurgery, Leiden University Medical Centre, Leiden, the Netherlands; c Division of Anaesthesia, Department of Medicine, University of Cambridge, Cambridge, United Kingdom; d The Golden Jubilee Spinal Cord Injury Centre, James Cook University Hospital, Middlesbrough, United Kingdom; e Department of Rehabilitation Medicine, Cambridge University Hospital NHS Foundation Trust, Cambridge, United Kingdom; f Anne McLaren Laboratory for Regenerative Medicine, Welcome Trust MRC Cambridge Stem Cell Institute, University of Cambridge, Cambridge, United Kingdom

**Keywords:** Traumatic brain injury, Spinal cord injury, Neurotrauma, Incidence, Mortality, Rehabilitation

## Abstract

**Introduction:**

The epidemiology and prognosis of the isolated traumatic brain injury (TBI) and spinal cord injury (SCI) are well studied. However, the knowledge of the impact of concurrent neurotrauma is very limited.

**Research questions:**

To characterize the longitudinal incidence of concurrent TBI and SCI and to investigate their combined impact on clinical care and outcomes, compared to a comparative but isolated SCI or TBI.

**Materials and methods:**

Data from 167,793 patients in the Trauma Audit and Research Network (TARN) registry collected in England and Wales between 2008 and 2018 were analysed. Tandem neurotrauma was defined as patients with concurrent TBI and SCI. The patient with isolated TBI or SCI was matched to the patient with tandem neurotrauma using propensity scores.

**Results:**

The incidence of tandem neurotrauma increased tenfold between 2008 and 2018, from 0.21 to 2.21 per 100,000 person-years. Patients in the tandem neurotrauma group were more likely to require multiple surgeries, ICU admission, longer ICU and hospital LOS, higher 30-day mortality, and were more likely to be transferred to acute hospitals and rehabilitation or suffer death at discharge, compared to patients with isolated TBI. Likewise, individuals with tandem neurotrauma compared to those with isolated SCI had a higher tendency to receive more than one surgery, ICU admission, longer LOS for ICU and higher mortality either at 30-day follow-up or at discharge.

**Discussion and conclusions:**

The incidence of tandem neurotrauma has increased steadily during the past decade. Its occurrence leads to greater mortality and care requirements, particularly when compared to TBI alone. Further investigations are warranted to improve outcomes in tandem neurotrauma.

## Highlights

• In this study, we conducted the first population-based cohort study (N = 167,784) to evaluate the incidence of tandem neurotrauma using data from the TARN registry between 2008 and 2018 in England and Wales. Further, the impact of tandem neurotrauma was investigated across a range of relevant clinical outcomes, using propensity matching with isolated TBI and SCI for individual characteristics and the severity of the injury. Together this demonstrates a rising incidence, and greater impact on individual and health delivery outcomes.

## 1 Introduction

Traumatic neurologic injuries, such as traumatic brain injury (TBI) and spinal cord injury (SCI), are significant public health concerns due to their associated mortality and morbidity. TBI generally refers to an acute brain injury resulting from mechanical energy to the head from external physical forces (Gardner and Zafonte, 2016) and conferring a transient or permanent impairment of function. In England and Wales, it is reported that 1.4 million patients per year attend hospital following head injuries, and TBI is the most common cause of death under the age of 40 years (Lawrence et al., 2016). Similarly, traumatic SCI leads to temporary or permanent dysfunction of the spinal cord (Ahuja et al., 2017). In the UK, the reported prevalence of SCI was approximately 40,000 out of about 64 million people, of which approximately 90% are caused by trauma (Palimaru et al., 2017).

However, TBI and SCI can coexist, and this has the potential to pose clinical challenges. For example, in the acute phase spinal precautions (such as cervical collars) may worsen intracranial pressure measures and complicate medical and nursing care within intensive care. Whilst the systemic perfusion pressure to the spinal cord and brain is shared, therapy is typically individualised for TBI as it is predicated on achieving intracranial pressure monitoring derived targets such as Cerebral Perfusion Pressure (Carney et al., 2017). Whilst evidence for the use of similar approaches in terms of monitoring of subdural intraspinal pressure at the injury site in SCI are emerging, these are not currently accepted as routine practice (Saadoun and Papadopoulos, 2016; Werndle et al., 2014). In the subacute phase of care, the UK specialist rehabilitation pathways are tailored to the pathologies in isolation but not combination: SCI rehabilitation is distinct from TBI rehabilitation pathways (although the principles of rehabilitation remains the same), and patients are often allocated to a rehabilitation unit specialising either in TBI or SCI, depending on which injury is deemed most severe (National Institute for Health and Care Excellence, 2016; National Institute for Health and Care Excellence: Clinical Guidance, 2014). The cognitive impairments caused by TBI can prevent smooth progress through conventional SCI programmes, therefore disability of patients from TBI can limit participation with SCI rehabilitation. Moreover, concurrent diagnoses can go undetected: severe TBI can preclude neurological assessment and identification of SCI, while mild TBI is often undiagnosed in the SCI population.

The incidence of concurrent TBI and SCI is, however, poorly characterised, with reports ranging from 12.5% to 74.2% (Pinto and Galang, 2016). This heterogeneity is partially due to methodologic differences including study design, inclusion criteria, sample size, and diagnostic criteria for both TBI and SCI (Bradbury et al., 2008). Ghobrial et al. demonstrated a steady increase in the incidence of concurrent TBI and SCI, from 0.82 per 100,000 hospital admissions in 1988 to 2.46 per 100,000 admissions in 2008 in the USA (Ghobrial et al., 2014). It is unclear whether this increase is also reflected in the UK or other regions of the world.

As such, whilst the epidemiology and prognosis of the isolated TBI and SCI are well studied, the knowledge of the impact of concurrent TBI and SCI is very limited (Sommer and Witkiewicz, 2004).

Given the limited research into the co-existing injuries, the objective of the present study was, firstly, to characterize the longitudinal incidence over a 10-year period, of the concurrent TBI and SCI which we have designated ‘Tandem Neurotrauma’. Secondly, we investigated the impact of tandem neurotrauma on relevant clinical outcomes, utilizing data from a nationwide trauma registry covering England and Wales.

## 2 Materials and methods

### 2.1 Study design and patients

An analysis of data captured by the Trauma Audit and Research Network (TARN) registry was conducted. All patients coded with either a TBI or SCI from England and Wales over 11 years (from 2008 to 2018) was extracted. TARN is a UK-based database collating trauma data from England, Wales, Ireland, and some hospitals from Continental Europe. Broadly TARN includes patients of any age, who arrive at the hospital alive after sustaining injury resulting in admission to hospital for three or more days, or require admission to intensive care or high dependency unit or interhospital transfer, or die from a traumatic injury. Isolated and minor injuries exist are excluded. These are prescriptively defined and included patients over 65 years with an isolated fracture of the femoral neck or pubic ramus or patients of age with single uncomplicated limb injuries. All patients from TARN in England and Wales are evaluated using a standardised protocol, and data are collated prospectively and systematically from the clinical presentation. Classification of trauma using the Abbreviated Injury Scale (AIS) is performed centrally. Details of TARN have been described previously (Lecky et al., 2002) and information is available at https://www.tarn.ac.uk.

Participating hospitals remove all patient identifiers and send data sheets to the TARN coordination centre at the University of Manchester. TARN holds approval from the UK Health Research Authority (section 251 PIAG) for analysis of the anonymised data for which it is the custodian.

### 2.2 Statistical analysis

‘Tandem neurotrauma’ was defined as patients with concurrent TBI and SCI. The incidence rates were calculated based on the annual mid-year populations of England and Wales obtained from the Office for National Statistics (https://www.ons.gov.uk), and subsequently expressed as cases per 100,000 persons-years with 95% confidence interval (CI). Additionally, the incidence rates analysis was carried out based on data provided by 35 hospitals that were ‘consistent submitters’ throughout the study period (core hospitals) (Moran et al., 2018) to consider any rise in incidence could simply be a consequence of increased case ascertainment from subsequently joining hospitals.

To create matched cohorts for comparison, a propensity score was used to match a patient in the isolated TBI group or the isolated SCI group to a patient in the tandem neurotrauma group. We used a multiple logistic regression model to create the propensity score for the probability of having a tandem neurotrauma, with the covariates of age (<30, 31–40, 41–50, 51–60, 61–70, 71–80, >80), gender, Glasgow Coma Score (GCS) (3–8, 9–12, 13–15), Injury Severity Score (ISS) band (1–8, 9–15 and > 15) and AIS (1–2, 3–4 and 5–6) for the severity of TBI or SCI. When matching a patient in the isolated SCI group to a patient in the tandem neurotrauma group, the level of SCI (cervical, thoracic and lumbar spinal cord) was used as an additional covariate. The probabilities from these models were used to generate a propensity score ranging from 0 to 1 for each patient. A nearest-neighbour 1:1 matching algorithm, without replacement, was applied based on the propensity score, with a calliper width of 0.005 standard deviation of the logit of the propensity score (Hemmila et al., 2010). The chosen calliper represents the maximum permitted difference between matched subjects. Two separate propensity matches were performed, one for patients who sustained isolated TBI or tandem neurotrauma, and one for patients who suffered either isolated SCI or tandem neurotrauma. Absolute standardised differences were computed to evaluate all covariates between tandem neurotrauma and those with isolated injury groups before and after matching, with a value of less than 10% and closer to zero indicating a more balanced cohort (Austin, 2009). The propensity score matching technique was chosen in the present study, as opposed to stratification or regression adjustment, because it is deemed as the closest approximate to a randomized controlled trial (RCT) for the purpose of our objective, with the greatest balance between treated and untreated cases (Austin et al., 2007). The outcomes were then compared between patients with tandem neurotrauma and those with isolated TBI or SCI. TARN contains limited detail on SCI or TBI specific disease characteristics or outcomes. Based on available information the length of stay (LOS) for hospital and intensive care unit (ICU), the number of operations, category of surgery, and mechanism of injury were compared to consider differences in injury, whilst survival or death at hospital discharge and on the 30th day, alongside discharge destination used as outcomes. Continuous variables were expressed as median and interquartile range (IQR), whereas categorical variables as number and percentage. Differences were examined using Wilcoxon rank sum test for continuous variables and the chi-square test for categorical variables.

For associations between tandem neurotrauma and discharge of destination, category of surgery and mechanism of injury, each category was dichotomised into binary outcome (e.g. discharge destination rehabilitation versus non-rehabilitation), and the number of operations was dichotomised into one surgery and more than one surgery.

Univariate logistic regression was used to estimate the odds ratio (OR) and 95% confidence interval of the outcomes and the mechanisms associated with the tandem versus isolated neurotrauma.

A two-sided P value of 0.05 was considered statistically significant. SPSS software, version 26.0 (SPSS, Inc., Chicago, IL, USA) was used for propensity score matching (Bertsekas and Tseng, 1988; Hansen, 2004; Hansen and Klopfer, 2006; Ho et al., 2007; Ho et al., 2011; Hansen and Bowers, 2008; Iacus et al., 2009; Thoemmes, 2012) and other statistical analyses.

## 3 Results

### 3.1 Demographics and the incidence of injuries

**Fig. 1:**
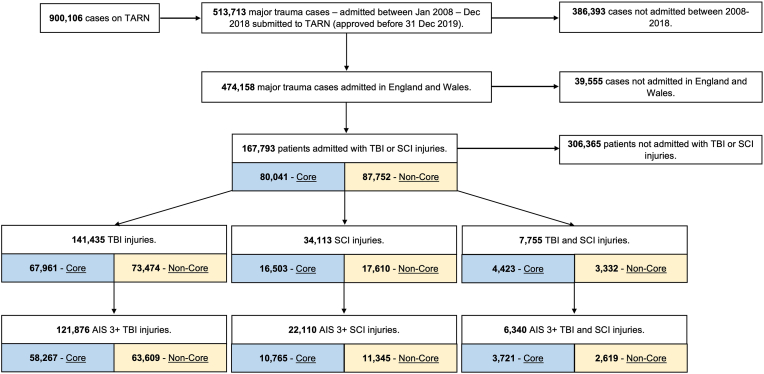
Strobe Diagram - Neuro trauma from 2008 to 2018.

Between 1 January 2008 to 31 December 2018, a total of 167,793 patients were reported with the diagnosis of TBI or SCI in England and Wales, of whom 141,435 patients suffered a TBI, 34,113 patients a SCI and 7755 patients tandem neurotrauma (Fig. 1).

**Fig. 2:**
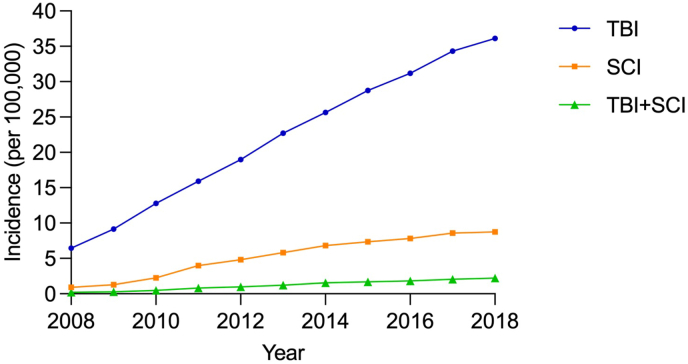
The incidence of TBI, SCI and tandem neurotrauma from 2008 to 2018.

**Fig. 3:**
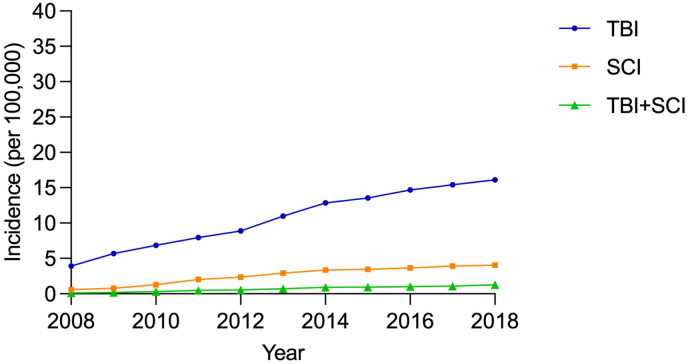
The incidence of TBI, SCI and tandem neurotrauma from 2008 to 2018 calculated based on 35 core hospitals.

The annual population-based incidence of TBI increased from 6.46 per 100,000 person-years (95% CI 6.24–6.67) in 2008 to 36.12 per 100,000 person-years (95% CI 35.64–36.61) in 2018, and the incidence of SCI increased from 0.92 (95% CI 0.84–1.00) to 8.76 (95% CI 8.53–9.00) per 100,000 person-years during the study period. Similarly, the incidence of tandem neurotrauma increased tenfold in 2018 compared to that of 2008, from 0.21 (95% CI 0.17–0.25) to 2.21 (95% CI 2.09–2.33) per 100,000 person-years (Fig. 2, Table S1). A similar trend was found when analysing incidence based on 35 core hospitals: the incidence of TBI increased from 3.91 per 100,000 person-years (95% CI 3.74–4.07) in 2008 to 16.13 per 100,000 person-years (95% CI 15.80–16.45) in 2018, and the incidence of SCI increased from 0.57 (95% CI 0.50–0.63) to 4.06 (95% CI 3.89–4.22) per 100,000 person-years. Likewise, the incidence of tandem neurotrauma increased tenfold in 2018 compared to that of 2008, from 0.13 (95% CI 0.10–0.16) to 1.27 (95% CI 1.18–1.36) per 100,000 person-years (Fig. 3, Table S2).

**Fig. 4:**
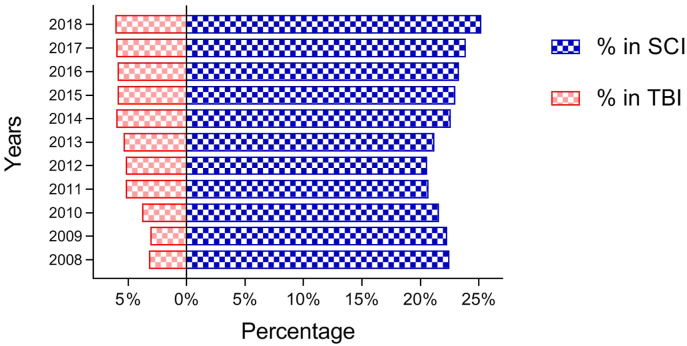
The percentage of tandem neurotrauma in patients with TBI and SCI.

During the study period, the proportion of patients with tandem neurotrauma increased slightly from 3.1% to 6.1% among TBI patients, while the proportion of patients with SCI increased from 20.6% to 25.2% during the last decade (Fig. 4). 29.8% of 141,435 patients with TBI were admitted to ICU, of whom 8.4% (3563) had concomitant SCI. While in the SCI group, 25.9% of 34,113 patients were admitted to ICU, of whom 40.4% (3563) had concomitant TBI.

### 3.2 The impact of tandem neurotrauma compared to isolated TBI

A total of 14,552 patients were matched for the tandem neurotrauma group and isolated TBI group (7276 patients for each group). The characteristics of the overall and matched cohort are shown in Table 1. As demonstrated in Table 2, 38.1% of 3182 patients in the tandem neurotrauma group received surgical treatment more than once, which was significantly higher than 26.7% of 2319 patients in the isolated TBI group (OR 1.68, 95% CI 1.50–1.89, P < 0.001). Surgery most commonly included spinal surgery in the tandem neurotrauma group (53.2%), followed by intracranial pressure (ICP) monitoring (10.6%) and craniectomy (4.8%). Whilst in the isolated TBI group, ICP monitoring was performed predominately (12.7%), followed by craniectomy (8.5%) and craniotomy (8.1%).

**Table 1:** Characteristics of the overall and matched cohorts and the standardized mean differences between groups.

	Overall Cohort (n = 141 435)			Matched Cohort (n = 14 552)		
	TBI (n = 133 684)	TBI + SCI (n = 7751)	SMD (%)	TBI (n = 7276)	TBI + SCI (n = 7276)	SMD (%)
Age (year)						
<30	25 751 (19.3)	1460 (18.8)	1.5	1316 (18.1)	1311 (18.0)	0.2
31-40	10 344 (7.7)	756 (9.8)	6.7	745 (10.2)	701 (9.6)	2.1
41-50	11 893 (8.9)	875 (11.3)	7.9	774 (10.6)	819 (11.3)	2.0
51-60	13 188 (9.9)	968 (12.5)	7.9	883 (12.1)	912 (12.5)	1.2
61-70	14 156 (10.6)	990 (12.8)	7.2	942 (12.9)	937 (12.9)	0.2
71-80	19 562 (14.6)	1109 (14.3)	0.4	1065 (14.6)	1058 (14.5)	0.3
>80	38 790 (29)	1593 (20.6)	21.5	1551 (21.3)	1538 (21.1)	0.4
Gender						
Female	48 602 (36.4)	2716 (35)	3.3	2583 (35.5)	2564 (35.2)	0.5
Male	85 082 (63.6)	5035 (65)		4693 (64.5)	4712 (64.8)	
GCS						
3-8	17 916 (14.8)	1301 (17.8)	7.9	1307 (18)	1295 (17.8)	0.4
9-12	11 880 (9.8)	529 (7.3)	9.9	517 (7.1)	522 (7.2)	0.3
13-15	91 005 (75.3)	5459 (74.9)	1.0	5452 (74.9)	5459 (75.0)	0.2
ISS Band						
1-8	5239 (3.9)	343 (4.4)	2.4	352 (4.8)	331 (4.5)	1.4
9-15	21 784 (16.3)	1963 (25.3)	21.0	1883 (25.9)	1895 (26.0)	0.4
>15	106 661 (79.8)	5445 (70.2)	21.1	5041 (69.3)	5050 (69.4)	0.3
AIS TBI severity						
1-2	16 311 (12.2)	3232 (41.7)	60.0	3114 (42.8)	3093 (42.5)	0.6
3-4	69 433 (51.9)	2860 (36.9)	30.7	2625 (36.1)	2678 (36.8)	1.5
5-6	47 940 (35.9)	1659 (21.4)	36.7	1537 (21.1)	1505 (20.7)	1.1

TBI: Traumatic brain injury.

SCI: Spinal cord injury.

SMD: Standardized mean difference.

GCS: Glasgow Coma Scale.

ISS: Injury Severity Score.

AIS: Abbreviated Injury Scale.

**Table 2:** Outcome between tandem neurotrauma group and isolated TBI group.

		TBI (n = 7276)	TBI + SCI (n = 7276)	P value	Odd ratio^a^	95% CI	P value
Length of stay		10 (5–21)	15 (7–33)	<0.001			
Admission to ICU		2393 (32.9)	3282 (45.1)	<0.001	1.677	1.568–1.793	<0.001
Length of stay for ICU		4 (2–11)	7 (2–18)	<0.001			
Mortality (30 days)		858 (11.8)	1336 (18.4)	<0.001	1.682	1.533–1.846	<0.001
Discharge destination							
Dead		898 (12.4)	1419 (19.5)	<0.001	1.720	1.570–1.883	<0.001
Other acute hospital		604 (8.3)	873 (12.0)	<0.001	1.505	1.349–1.679	<0.001
Home (own)		4259 (58.7)	3154 (43.4)	<0.001	0.541	0.506–0.577	<0.001
Home (with support)^b^		249 (3.4)	191 (2.6)	0.005	0.760	0.628–0.921	0.005
Rehabilitation		700 (9.6)	1244 (17.1)	<0.001	1.936	1.754–2.138	<0.001
Nursing home		331 (4.6)	189 (2.6)	<0.001	0.559	0.466–0.671	<0.001
Other^c^		219 (3.0)	195 (2.7)	0.229			
Operation							
>1 operation		620 (26.7)	1211 (38.1)	<0.001	1.684	1.498–1.892	<0.001
1 operation		1699 (73.3)	1971 (61.9)				
Surgery							
ICP monitoring		328 (12.7)	357 (10.6)	0.011	0.814	0.694–0.954	0.011
Spine surgery^d^		60 (2.3)	1794 (53.2)	<0.001	47.745	36.638–62.220	<0.001
Craniotomy		209 (8.1)	90 (2.7)	<0.001	0.311	0.242–0.401	<0.001
Craniectomy		220 (8.5)	163 (4.8)	<0.001	0.545	0.442–0.672	<0.001
Drain of ventricle		55 (2.1)	66 (2.0)	0.639			
Mechanism							
Fall>2 m		1357 (18.7)	2412 (33.2)	<0.001	2.163	2.004–2.335	<0.001
Fall<2 m		2919 (40.1)	2015 (27.7)	<0.001	0.572	0.533–0.613	<0.001
Road traffic collision		2152 (29.6)	2521 (34.6)	<0.001	1.262	1.177–1.354	<0.001
Penetrating^e^		117 (1.6)	27 (0.4)	<0.001	0.228	0.150–0.347	<0.001
Other^f^		731 (10.0)	301 (4.1)	<0.001	0.386	0.336–0.444	<0.001

TBI: Traumatic brain injury; SCI: Spinal cord injury; CI: Confidence interval; ICU: Intensive care unit.

a Univariate analysis.

b Home (relative or other carer) + Social care.

c No fixed abode + Alive at 30 days still in hospital + Other institution + Not known.

d Fixation of spine + Fusion of spine + Spinal stabilisation.

e Shooting + Stabbing.

f Blast + Blows + Burn + Crush + Other.

Patients with tandem neurotrauma were more likely to be admitted to ICU (OR 1.68, 95% CI 1.57–1.79, P < 0.001), and had longer ICU LOS (7 versus 4 days, P < 0.001) as well as longer hospital LOS (15 versus 10 days, P < 0.001) compared to patients in the isolated TBI group. Similarly, the 30-day mortality of the tandem neurotrauma group was significantly higher than that of the isolated TBI group (OR 1.68, 95% CI 1.53–1.85, P < 0.001). Regarding discharge destination, those with tandem neurotrauma were more likely to be transferred to other acute hospitals (OR 1.51, 95% CI 1.35–1.68 P < 0.001) and rehabilitation facilities (OR 1.94, 95% CI 1.75–2.14, P < 0.001) or suffer death at the point of hospital discharge (OR 1.72, 95% CI 1.57–1.88, P < 0.001), with fewer patients returning home with (OR 0.76, 95% CI 0.63–0.92, P = 0.005) or without support (OR 0.54, 95% CI 0.51–0.58, P < 0.001), compared to the isolated TBI group. Falling from a height more than 2 m and road traffic collisions were identified as risk factors for tandem neurotrauma (OR 2.16, 95% CI 2.00–2.34, P < 0.001 and OR 1.26, 95% CI 1.18–1.35, P < 0.001, respectively).

### 3.3 The impact of tandem neurotrauma compared to isolated SCI

A total of 11,852 subjects were matched for patients with tandem neurotrauma and patients with isolated SCI (5926 patients for each group). The characteristics of the overall and matched cohorts are shown in Table 3, while the differences between these cohorts are shown in Table 4. Of the 2518 patients who received surgical treatment in the tandem neurotrauma group, 34.4% of them underwent more than one surgery, which was significantly higher than 31.5% of 2695 patients in the isolated SCI group (OR 1.14, 95% CI 1.02–1.28, P = 0.027). In the tandem neurotrauma group, spinal surgery was the most frequent (59.2%) surgery performed, followed by ICP monitoring (5.2%) and craniectomy (2.5%). Similarly, and as expected, spinal surgery was also performed predominately (64.9%) for patients with isolated SCI, followed by ICP monitoring (0.3%).

**Table 3:** Characteristics of the overall and matched cohorts and the standardized mean differences between groups.

	Overall Cohort (n = 34 100)			Matched Cohort (n = 11 852)		
	SCI (n = 26 349)	TBI + SCI (n = 7751)	SMD (%)	SCI (n = 5926)	TBI + SCI (n = 5926)	SMD (%)
Age (year)						
<30	4337 (16.5)	1460 (18.8)	4.4	884 (14.9)	878 (14.8)	0.3
31-40	2475 (9.4)	756 (9.8)	0.9	535 (9.0)	535 (9.0)	<0.1
41-50	3016 (11.4)	875 (11.3)	0.5	647 (10.9)	663 (11.2)	0.9
51-60	3883 (14.7)	968 (12.5)	6.4	784 (13.2)	786 (13.3)	0.1
61-70	3808 (14.5)	990 (12.8)	3.9	813 (13.7)	831 (14.0)	0.9
71-80	3952 (15.0)	1109 (14.3)	1.4	947 (16.0)	914 (15.4)	1.6
>80	4878 (18.5)	1593 (20.6)	5.2	1316 (22.2)	1319 (22.3)	0.1
Gender						
Female	11 199 (42.5)	2716 (35.0)	15.6	2148 (36.2)	2174 (36.7)	0.9
Male	15 150 (57.5)	5035 (65.0)		3778 (63.8)	3752 (63.3)	
GCS						
3-8	461 (1.9)	1301 (17.8)	41.6	378 (6.4)	381 (6.4)	0.1
9-12	381 (1.6)	529 (7.3)	21.8	249 (4.2)	253 (4.3)	0.3
13-15	23 072 (96.5)	5459 (74.9)	49.8	5299 (89.4)	5292 (89.3)	0.3
ISS Band						
1-8	3816 (14.5)	343 (4.4)	47.6	318 (5.4)	330 (5.6)	1.0
9-15	14 241 (54.0)	1963 (25.3)	64.6	1911 (32.2)	1883 (31.8)	1.1
>15	8292 (31.5)	5445 (70.2)	83.0	3697 (62.4)	3713 (62.7)	0.6
AIS SCI severity						
1-2	8714 (33.1)	3288 (42.4)	18.9	2458 (41.5)	2450 (41.3)	0.3
3-4	16 350 (62.1)	3902 (50.3)	23.2	3067 (51.8)	3063 (51.7)	0.1
5-6	1285 (4.9)	561 (7.2)	8.9	401 (6.8)	413 (7.0)	0.8
Level of SCI						
Cervical spine	5649 (21.4)	2321 (29.9)	18.9	1765 (29.8)	1765 (29.8)	<0.1
Thoracic spine	5761 (21.9)	1655 (21.4)	1.6	1260 (21.3)	1275 (21.5)	0.6
Lumbar spine	6541 (24.8)	707 (9.1)	55.5	562 (9.5)	577 (9.7)	0.9

SCI: Spinal cord injury; TBI: Traumatic brain injury; SMD: Standardized mean difference; GCS: Glasgow Coma Scale; ISS: Injury Severity Score.

AIS: Abbreviated Injury Scale.

**Table 4:** Outcome between tandem neurotrauma group and isolated SCI group.

		SCI (n = 5926)	TBI + SCI (n = 5926)	P value	Odd ratio^a^	95% CI	P value
Length of stay		15 (7–30)	15 (7–31)	0.459			
Admission to ICU		2020 (34.1)	2245 (37.9)	<0.001	1.179	1.094–1.271	<0.001
Length of stay for ICU		5 (2–13)	6 (2–17)	0.001			
Mortality (30 days)		619 (10.4)	762 (12.9)	<0.001	1.265	1.130–1.416	<0.001
Discharge destination							
Dead		657 (11.1)	826 (14.0)	<0.001	1.299	1.165–1.450	<0.001
Other acute hospital		780 (13.2)	710 (12.0)	0.053			
Home (own)		3013 (50.9)	2887 (48.8)	0.022	0.919	0.855–0.988	0.022
Home (with support)^b^		148 (2.5)	159 (2.7)	0.523			
Rehabilitation		980 (16.6)	990 (16.7)	0.799			
Nursing home		193 (3.3)	172 (2.9)	0.266			
Other^c^		148 (2.5)	173 (2.9)	0.156			
Operation							
>1 operation		848 (31.5)	865 (34.4)	0.027	1.140	1.015–1.279	0.027
1 operation		1847 (68.5)	1653 (65.6)				
Surgery							
ICP monitoring		8 (0.3)	139 (5.2)	<0.001	19.592	9.588–40.034	<0.001
Spine surgery^d^		1847 (64.9)	1572 (59.2)	<0.001	0.784	0.703–0.875	<0.001
Craniotomy		1 (0.04)	54 (2.0)	<0.001	59.045	8.163–427.110	<0.001
Craniectomy		1 (0.04)	67 (2.5)	<0.001	73.628	10.214–530.740	<0.001
Drain of ventricle		1 (0.04)	33 (1.2)	<0.001	35.794	4.892–261.891	<0.001
Mechanism							
Fall>2 m		1547 (26.1)	2038 (34.4)	<0.001	1.484	1.371–1.606	<0.001
Fall<2 m		2316 (39.1)	1807 (30.5)	<0.001	0.684	0.634–0.738	<0.001
Road traffic collision		1798 (30.3)	1823 (30.8)	0.618			
Penetrating^e^		33 (0.6)	21 (0.4)	0.102			
Other^f^		232 (3.9)	237 (4.0)	0.814			

TBI: Traumatic brain injury; SCI: Spinal cord injury; CI: Confidence interval; ICU: Intensive care unit.

a Univariate analysis.

b Home (relative or other carer) + Social care.

c No fixed abode + Alive at 30 days still in hospital + Other institution + Not known.

d Fixation of spine + Fusion of spine + Spinal stabilisation.

e Shooting + Stabbing.

f Blast + Blows + Burn + Crush + Other.

Patients with tandem neurotrauma were more likely to be admitted to ICU (OR 1.18, 95% CI 1.09–1.27, P < 0.001), and had longer ICU LOS (6 versus 5 days, P = 0.001) compared to patients in the isolated SCI group, whilst hospital LOS was comparable between the groups (Table 4). The 30-day mortality of patients with tandem neurotrauma was significantly higher than that of patients with isolated SCI (OR 1.27, 95% CI 1.13–1.42, P < 0.001). Patients with tandem neurotrauma were more likely to die (OR 1.30, 95% CI 1.17–1.45, P < 0.001) and fewer patients returned home without support (OR 0.92, 95% CI 0.86–0.99, P = 0.022). Patients indexed as discharged to other acute hospitals, rehabilitation, nursing home, or their own home with support were comparable between the two groups. Falling from a height more than 2 m was also identified as a risk factor for tandem neurotrauma (OR 1.48, 95% CI 1.37–1.61, P < 0.001).

## 4 Discussion

This is the first population-based cohort study to evaluate the incidence and impact of tandem neurotrauma in the UK. Using data from the TARN registry the annual incidence of tandem neurotrauma has increased tenfold between 2008 and 2018 in England and Wales. When matched with isolated TBI and SCI using propensity scores relating to individual characteristics and the severity of the injury, patients with tandem neurotrauma had a higher risk of mortality compared to isolated TBI and SCI patients. In comparison with isolated TBI, the presence of tandem neurotrauma clearly impacts on the clinical management leading to an increased risk of ICU admission, longer ICU LOS, need for multiple surgical procedures, and on-going treatment requiring transfer to an acute hospital or rehabilitation centre at discharge. Identified risk factors included falls from a height more than 2 m and road traffic collisions, suggestive of higher impact injuries.

Previous reports on the incidence of tandem neurotrauma have varied widely, ranging from 12.5% to 74.2% (Pinto and Galang, 2016) with estimates from population-based research lacking. The result in the current study indicates a steady increase in the incidence of tandem neurotrauma over the past decade, which is in line with the only previous similar study in the USA. They reported the incidence rose from 0.82 per 100,000 hospital admissions to 2.46 per 100,000 hospital admissions over a 20-year period in the USA (Ghobrial et al., 2014). Similarly, the present study found that the incidence of both tandem neurotrauma and isolated SCI increased approximately tenfold over the study period. This is compatible with the findings of Ghobrial et al. who found that the rise of tandem neurotrauma was mirrored by a rise in the incidence of SCI (Ghobrial et al., 2014). The reason for the observed substantial increase in TBI, SCI and tandem neurotrauma remains elusive. Improved case ascertainment is likely one contributing factor. Although in our sensitivity analysis increases were consistent amongst the original TARN centres, other unmeasured healthcare factors may have contributed. For example imaging rates have increased in the UK following the implementation of NICE clinical guidelines for suspected head injury from 2007 to 2014 improving brain injury detection. Other factors may include population aging and co-morbidity.

Nevertheless these uncertainties will not have limited the comparison of tandem neurotrauma with isolated neurotrauma. Previous literature indicated that the combination of TBI and SCI leads to inferior outcomes following SCI, which includes clinical and functional outcomes, as well as a less efficient rehabilitation process (Macciocchi et al., 2004, 2012). However, these studies are limited by a relatively small number of participants. The impact of tandem neurotrauma compared to isolated TBI is largely unknown. Unfortunately, the TARN registry is limited with regards to outcome data. Nevertheless, all reported outcomes differed significantly when compared to either isolated injury: mortality, ICU admission, ICU LOS and the number of operations. The additional significantly differences in hospital LOS and discharge destination were even found when compared with isolated TBI.

The basis for any disparity in functional outcomes between tandem neurotrauma and TBI versus SCI has not been explored in detail. Neurological deficits as a consequence of SCI and TBI are likely to result in more disability than expected, and exceed that anticipated from the combination of identical isolated brain and spinal cord injury (Inoue et al., 2013).

In our matched cohorts, patients with tandem neurotrauma were more likely to undergo a procedure associated with global rises in ICP (craniectomy or placement of an external ventricular drain) versus an isolated mass lesion (craniotomy). This could be reflective of higher impact injuries in the tandem injury group, one of the identified risks factors. In addition, secondary injury mechanisms may also contribute to worse outcomes. Both TBI and SCI trigger an elevated inflammatory state (Pinto and Galang, 2016), which contributes to morbidity and adverse outcome, e.g. through microglia activation, increase of cytokines IL-1, IL-6 and TNF-a, and eventual neural and glial apoptosis (Profyris et al., 2004; Donnelly and Popovich, 2008; Zhang and Gensel, 2014; Witcher et al., 2015). The extent of inflammatory responses may differ between SCI and TBI, e.g. with regard to macrophage/microglia activation, neutrophil recruitment, and accumulation of B and T cells (Zhang and Gensel, 2014). Animal studies have also found that inflammation and degeneration were induced within the brain following SCI, resulting in long-term cognitive deficits (Wu et al., 2014). It is likely that in tandem neurotrauma this is aggravated.

The strong association between the tandem neurotrauma and mortality demonstrated in this study is consistent with the previous reports. In a retrospective study, Varma et al. found the presence of concomitant TBI was significantly associated with early mortality (OR 3.7, 95% CI 2.2–6.0, P < 0.0001) (Varma et al., 2010), which is similar to our findings in the present study (OR 1.68, 95% CI 1.53–1.85, P < 0.001). Moreover, we additionally reported that the presence of concurrent SCI was also associated with higher 30-day mortality (OR 1.27, 95% CI 1.13–1.42, P < 0.001). Our study findings are based on a much larger patient cohort and strengthen previous findings concerning the influence of combined injuries on mortality. Interestingly, despite motor vehicle collisions were previously identified as the risk factor for tandem neurotrauma, it was only confirmed when compared with isolated TBI patients in this study, whilst a fall over 2 m was associated with tandem neurotrauma no matter comparing isolated TBI or SCI.

Nott et al. performed a cross-sectional, case-matched study comparing medical and functional outcomes between tandem neurotrauma, isolated TBI and SCI, which demonstrated that LOS for rehabilitation was significantly longer in SCI and tandem neurotrauma patients, both of which required more daily care and support than TBI only patients, whilst patients with tandem neurotrauma received similar levels of care and support to those with SCI (Nott et al., 2014). This is consistent with our findings, pointing towards the importance of the SCI with regards to a patient's rehabilitation requirements following tandem neurotrauma.

Although rehabilitation for TBI and SCI, is a vital part of gold-standard care (Maas et al., 2017), there are important challenges for its delivery, such as the complex needs of head-injured patients, resource limitation, commissioning issues and lack of evidence/research (Seeley and Hutchinson, 2006; Wright et al., 2016; Christodoulou et al., 2019). Furthermore, in the UK, SCI and TBI rehabilitation pathways are distinct, with few centres (mainly hyperacute rehabilitation units) catering specifically for both pathologies. Given these pathologies can have significantly differing requirements, and the increasing population burden revealed here, we deem it important to address this knowledge gap.

### 4.1 Limitations

The data presented is based on England and Welsh major trauma patients within the TARN registry. It is recognised that patient recruitment is less than 100%, with internal evaluations during this study period highlighting TARN received data of patient from 60% to 100% of all NHS trauma receiving hospitals in England and about 70% of all trauma receiving hospitals in Wales (Lecky et al., 2015). Whilst TARN would therefore have been unable to capture the true incidence of neurotrauma, this is unlikely to have influenced the comparative analysis of SCI, TBI and tandem neurotrauma. Further the analysis is also limited to the type of data, including choice of classification systems and outcomes, used by TARN. Whilst this places limitations on the more detailed characterisation or long-term outcomes of the cohorts, including sub-groups, overall, as the first study to investigate the impact of tandem neurotrauma within a large number of patients, based on co-variates that reflect the known major determinants of outcome in these populations, the data presented here provides a valuable contribution and foundation for further investigation.

## 5 Conclusions

The incidence of tandem neurotrauma captured in TARN has increased steadily during the past decade in line with increases in both TBI and SCI patients. Tandem neurotrauma leads to increased mortality and additional care requirements. The present uncertainty behind the determinants of outcome and the additional care requirements of tandem neurotrauma warrants further research aiming to identify specific patient needs, risk factors and rehabilitation strategies.

## Funding

XY is supported by Cultural Foundation Grant (Award number 40026482) from Prins Bernhard Cultural Foundation in the Netherlands during the conduct of the study. The grant has no role in the conduction of this research.

## Declaration of competing interest

The authors declare that they have no known competing financial interests or personal relationships that could have appeared to influence the work reported in this paper.
