# Supplementary material for: The incidence and impact of ‘Tandem Neurotrauma’
Source: Brain Spine. 2023 Oct 21;3:102702. doi: 10.1016/j.bas.2023.102702 (PMC10668105; doi:10.1016/j.bas.2023.102702)
Supplement: Multimedia component 1 [file mmc1.docx]

Table S1 Incidence of TBI, SCI and tandem neurotrauma

| Year | TBI | Incidence (/100 000) | 95% CI | SCI | Incidence (/100 000) | 95% CI | TBI+  SCI | Incidence (/100 000) | 95% CI | Total number of injury | Population of England and Wales ^a^ |
| --- | --- | --- | --- | --- | --- | --- | --- | --- | --- | --- | --- |
| 2008 | 3 541 | 6.46 | 6.24-6.67 | 507 | 0.92 | 0.84-1.00 | 114 | 0.21 | 0.17-0.25 | 3 934 | 54 841 700 |
| 2009 | 5 048 | 9.14 | 8.89-9.39 | 700 | 1.27 | 1.17-1.36 | 156 | 0.28 | 0.24-0.33 | 5 592 | 55 235 300 |
| 2010 | 7 116 | 12.78 | 12.48-13.07 | 1 246 | 2.24 | 2.11-2.36 | 269 | 0.48 | 0.43-0.54 | 8 093 | 55 692 400 |
| 2011 | 8 949 | 15.93 | 15.60-16.26 | 2 235 | 3.98 | 3.81-4.14 | 463 | 0.82 | 0.75-0.90 | 10 721 | 56 170 900 |
| 2012 | 10 733 | 18.97 | 18.61-19.33 | 2 715 | 4.80 | 4.62-4.98 | 559 | 0.99 | 0.91-1.07 | 12 889 | 56 567 800 |
| 2013 | 12 925 | 22.70 | 22.30-23.09 | 3 305 | 5.80 | 5.61-6.00 | 702 | 1.23 | 1.14-1.32 | 15 528 | 56 948 200 |
| 2014 | 14 728 | 25.65 | 25.24-26.07 | 3 915 | 6.82 | 6.61-7.03 | 886 | 1.54 | 1.44-1.64 | 17 757 | 57 408 700 |
| 2015 | 16 642 | 28.75 | 28.31-29.19 | 4 258 | 7.36 | 7.13-7.58 | 979 | 1.69 | 1.59-1.80 | 19 921 | 57 885 400 |
| 2016 | 18 195 | 31.17 | 30.71-31.61 | 4 565 | 7.82 | 7.59-8.05 | 1 065 | 1.82 | 1.71-1.93 | 21 695 | 58 381 200 |
| 2017 | 20 162 | 34.32 | 33.85-34.80 | 5 032 | 8.57 | 8.33-8.80 | 1 205 | 2.05 | 1.94-2.17 | 23 989 | 58 744 600 |
| 2018 | 21 354 | 36.12 | 35.64-36.61 | 5 181 | 8.76 | 8.53-9.00 | 1 304 | 2.21 | 2.09-2.33 | 25 231 | 59 115 800 |

^a^ Annual mid-year populations of England and Wales obtained from the Office for National Statistics (https://www.ons.gov.uk)

TBI: Traumatic brain injury; SCI: Spinal cord injury; CI: Confidence interval

Table S2 Incidence of TBI, SCI and tandem neurotrauma calculated based on 35 core hospitals

| Year | TBI | Incidence (/100 000) | 95% CI | SCI | Incidence (/100 000) | 95% CI | TBI+  SCI | Incidence (/100 000) | 95% CI | Total number of injury | Population of England and Wales ^a^ |
| --- | --- | --- | --- | --- | --- | --- | --- | --- | --- | --- | --- |
| 2008 | 2 142 | 3.91 | 3.74-4.07 | 310 | 0.57 | 0.50-0.63 | 71 | 0.13 | 0.10-0.16 | 2 381 | 54 841 700 |
| 2009 | 3 142 | 5.69 | 5.49-5.89 | 434 | 0.79 | 0.71-0.86 | 97 | 0.18 | 0.14-0.21 | 3 479 | 55 235 300 |
| 2010 | 3 820 | 6.86 | 6.64-7.08 | 721 | 1.29 | 1.20-1.39 | 178 | 0.32 | 0.27-0.37 | 4 363 | 55 692 400 |
| 2011 | 4 460 | 7.94 | 7.71-8.17 | 1 131 | 2.01 | 1.90-2.13 | 274 | 0.49 | 0.43-0.55 | 5 317 | 56 170 900 |
| 2012 | 5 019 | 8.87 | 8.63-9.12 | 1 329 | 2.35 | 2.22-2.48 | 313 | 0.55 | 0.49-0.61 | 6 035 | 56 567 800 |
| 2013 | 6 256 | 10.99 | 10.71-11.26 | 1 665 | 2.92 | 2.78-3.06 | 410 | 0.72 | 0.65-0.79 | 7 511 | 56 948 200 |
| 2014 | 7 377 | 12.85 | 12.56-13.14 | 1 925 | 3.35 | 3.20-3.50 | 519 | 0.90 | 0.83-0.98 | 8 783 | 57 408 700 |
| 2015 | 7 838 | 13.54 | 13.24-13.84 | 1 999 | 3.45 | 3.30-3.60 | 547 | 0.94 | 0.87-1.02 | 9 290 | 57 885 400 |
| 2016 | 8 569 | 14.68 | 14.37-14.99 | 2 136 | 3.66 | 3.50-3.81 | 602 | 1.03 | 0.95-1.11 | 10 103 | 58 381 200 |
| 2017 | 9 061 | 15.42 | 15.11-15.74 | 2 300 | 3.92 | 3.76-4.08 | 640 | 1.09 | 1.01-1.17 | 10 721 | 58 744 600 |
| 2018 | 9 533 | 16.13 | 15.80-16.45 | 2 398 | 4.06 | 3.89-4.22 | 749 | 1.27 | 1.18-1.36 | 11 182 | 59 115 800 |

^a^ Annual mid-year populations of England and Wales obtained from the Office for National Statistics (https://www.ons.gov.uk)

TBI: Traumatic brain injury; SCI: Spinal cord injury; CI: Confidence interval
